# The ampulla of Vater: A potential target for metastatic melanoma?

**DOI:** 10.1093/jscr/rjac621

**Published:** 2023-01-18

**Authors:** David Armany, Preet Gosal, Stuart Adams

**Affiliations:** General Surgery Department, Nepean Hospital, NBMLHD, NSW, Australia; General Surgery Department, Nepean Hospital, NBMLHD, NSW, Australia; Department of Anatomical Pathology, NSW Health Pathology, Nepean Hospital, NBMLHD, NSW, Australia

**Keywords:** metastatic, melanoma, ampulla of Vater, obstructive jaundice, acute pancreatitis

## Abstract

Malignant melanomas are aggressive cancers that can prove to be fatal, with Australia harbouring the highest incidence of skin cancers worldwide. Surprisingly, as little as 13.4% of patients undergoing surgical resection of high-risk melanomas remain disease-free after 2 years, with 31.6% showing evidence of distant spread. Although rare, secondary tumours of the ampulla of Vater have been documented, with the most common primaries involving breast, renal and melanoma cancers. We report the case of a malignant melanoma of the ampulla of Vater occurring in a patient 4-year post-surgical resection of a Stage II melanoma manifesting as acute pancreatitis with obstructive jaundice. Given the rarity of secondary ampullary tumours, metastatic melanoma should always be considered in patients with obstructive jaundice and a history of melanoma resection. In the absence of distant disease, surgery may be considered after consensus at multidisciplinary team meetings and after consideration of the patient preference.

## INTRODUCTION

Malignant melanomas are insidious aggressive cancers that can prove to be fatal, with Australia harbouring the highest incidence of skin cancers worldwide [1]. Melanomas occur as a malignant transformation of melanocytes, resulting in amplification of growth factors and insensitivity to growth inhibitors among other features, perpetuating tissue invasion and metastases [2]. Several environmental and genetic risk factors play a role, including exposure to ultraviolet B radiation, fair complexion and numerous atypical naevi [3].

The diagnosis of primary melanoma is often discovered incidentally by patients or their primary care physician as a new skin lesion that may be increasing in size, bleeding or itchy. Subsequent skin excision and histopathology confirm the diagnosis, and if captured early, 90% may be curable by surgical resection [1]. Here, we present a case of a 75-year-old male presenting with obstructive jaundice and acute pancreatitis treated with endoscopic retrograde cholangiopancreatography (ERCP) and stenting with a history of prior primary melanoma excision.

## CASE REPORT

A 75-year-old Vietnam veteran with a background history of Type II diabetes, ischaemic heart disease, hypertension, obstructive sleep apnoea and alcoholism was referred to our institution with jaundice and epigastric abdominal pain.

On further evaluation, the patient reported a significant surgical history, including a previous primary nodular melanoma excised from left upper back in 2017 (Breslow thickness: 3 mm), along with coronary artery bypass grafting in 2018. He also disclosed unintentional weight loss of 20 kg over 3 months and anorexia along with intermittent epigastric abdominal pain for the last 11 months. The patient was overtly jaundiced on clinical examination with scleral icterus and mild focal epigastric tenderness. There was no palpable lymphadenopathy or abdominal mass.

His biochemical analysis revealed an obstructive LFT pattern with a bilirubin of 119μmol/l an Amylase of 127 U/l and Lipase of 675 U/l along with an elevated CEA 5.7 μg/l and CA19.9131 IU/ml. Additionally, the patient had an acute kidney injury (AKI) with a Cr of 236 μmol/l and an eGFR of 23 ml/min/1.73 m^2^.

**Figure 1:**
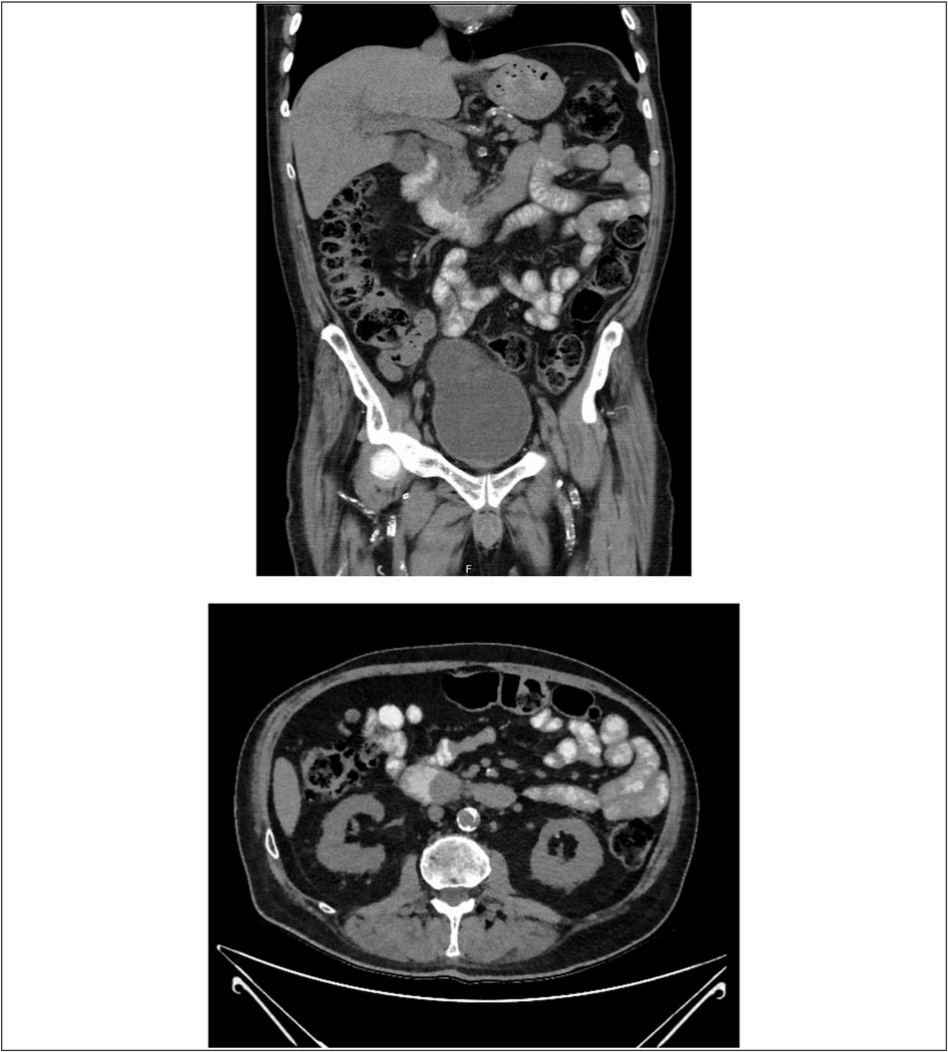
CT with oral contrast revealing a soft tissue ampullary lesion on axial and coronal images.

A computed tomography (CT) with oral contrast of the patient’s abdomen and pelvis revealed a soft tissue ampullary lesion projecting into the duodenal lumen and resultant dilatation of the CBD and intrahepatic ducts. Adequate assessment of lesion was limited by the lack of IV contrast in the context of the patients AKI. Additionally, the periportal, intraabdominal and epicardial lymph nodes were mildly prominent (Fig. 1).

**Figure 2:**
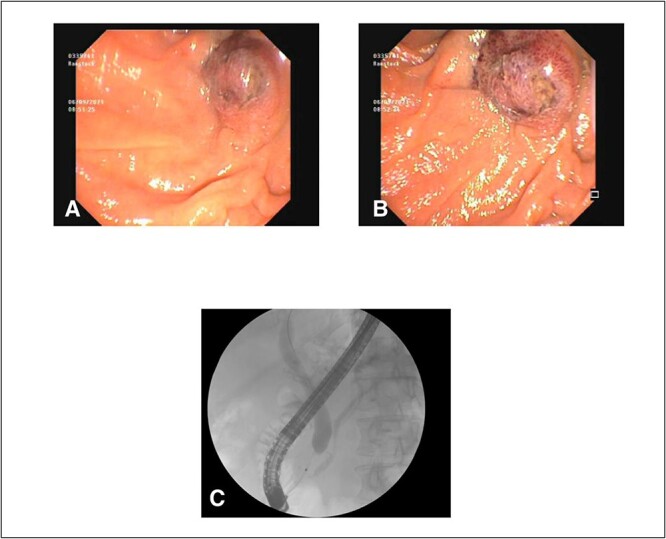
(**A**) and (**B**) malignant appearing soft tissue infiltrative mass within the ampulla of Vater seen on ERCP; (**C**) cholangiopancreatography showing stenosed distal third of the common bile duct along with a patent pancreatic duct.

**Figure 3:**
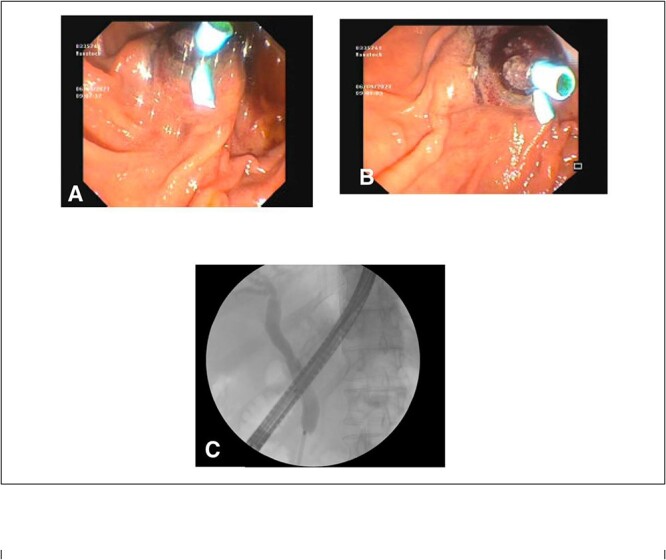
(**A**) and (**B**) endoscopic images showing a single plastic stent inserted into the distal CBD, protruding into the second part of the duodenum; (**C**) position confirmed on cholangiopancreatography revealing resolution of distal CBD filling defect.

After consultation with our Hepatobiliary service, the consensus was for the patient to undergo an urgent ERCP. Intraoperatively, a duodenoscope was introduced through the mouth and was advanced to the duodenum at the ampulla of Vater. The major papillae were bulging and a malignant appearing infiltrative mass was visualized. A tapered tip cannula was inserted through the ampulla and contrast was injected, revealing a patent PD and a single 18-mm long stenosis of the distal third of the CBD with significant dilatation of the proximal CBD and intrahepatic ducts (Fig. 2). A 10Fr 7-cm transpapillary plastic stent was inserted with a single internal flap, 5.5 cm, into the CBD. The biliary pancreatic junction was biopsied with cold forceps for histology. Cholangiopancreatography revealed the stent was in good position with good flow of contrast into the duodenum beyond the filling defect (Fig. 3).

**Figure 4:**
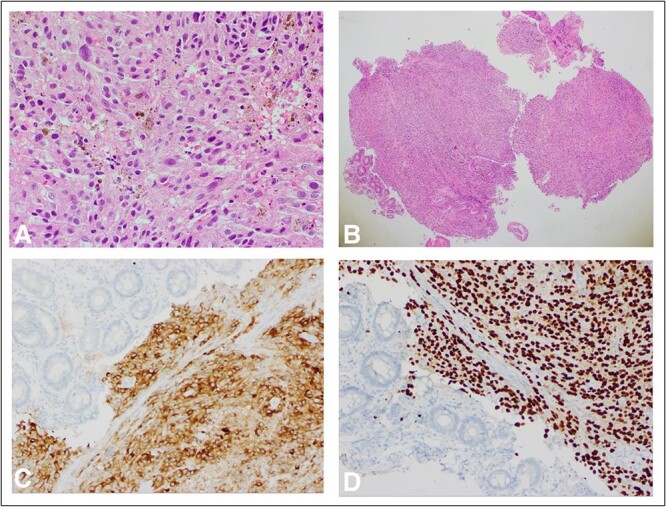
(**A**) H&E ×400 slide showing pigmented melanoma cells with surrounding normal tissue; (**B**) H&E ×40 slide with normal ampulla tissue on far left and tumour in centre; (**C**) MelanA ×200 stain with melanoma; and adjacent normal ampullary tissue on the left; cytoplasmic expression of MelanA/MART1 highlights how the melanocytes are obscured by a dense lymphocytic infiltrate than in the conventional H&E slide; (**D**) Sox10 stain ×200 similarly showing discoloration by malignant melanoma cells with surrounding normal ampullary tissue.

Histopathology of the ampullary lesions collected at ERCP showed evidence of malignant large cell tumour with diffuse and focally nested proliferation of epithelioid-shaped cells positive for Melan-A and Sox10 on immunohistochemistry, which was consistent with malignant melanoma (Fig. 4). Additionally. tumour cells were positive for BRAF v600 mutations; thus, this patient would have been eligible for BRAF inhibitors.

Following the histopathology results, the patient underwent a staging CT brain revealing multiple haemorrhagic cerebral metastases compatible with metastatic melanoma. He was discussed at the UGI multidisciplinary team (MDT) meeting and consensus was reached to refer this patient for palliative and supportive care.

## DISCUSSION

Secondary tumours of the ampulla of Vater are a rare phenomenon, and as such, no case series or large-scale studies have been done; however, prior literature reviews have revealed common primaries to include breast, renal and melanomas [4]. In the context of melanomas, metastasis to the gastrointestinal tract has been well established with the stomach and small intestine occurring with greater frequency in comparison to the oesophagus, duodenum and large colon regardless of the primary melanomas anatomic site [5]. These patients symptoms tend to manifest as either dysphagia, upper gastrointestinal bleeding, obstructive jaundice or bowel obstruction as a result of intussusception [6].

Notably, this patient had a significant history of previous primary, Stage II melanoma resection of his left upper back in 2017 with curative surgical margins. The patient did not undergo any adjuvant therapy. Surprisingly, only a minority of patients post-resection of high-risk primary melanomas remain disease-free at 2 years, with a large percentage developing distant metastatic disease [7]. Currently, only low-level evidence exists for the consensus regarding follow-up for Stage II melanomas, which involves biannual skin examinations for 2 years than annually for a following of 8 years [8, 9]. Although our patient was not compliant with routine follow-ups, the effect this would have had on his overall survival is unclear. Given the rarity of secondary ampullary tumours, metastatic melanoma should be considered in patients with obstructive jaundice secondary to a radiologically evident lesion and a history of melanoma resection.

Once identified, these patients should be discussed at the next available MDT meeting with available staging scans to assess for further distant metastatic disease. Although uncommon, pancreaticoduodenectomy has been used to treat solitary metastatic melanoma in the ampulla with a palliative intent due to the probability of hidden metastatic disease elsewhere [10]. However, data on this are extremely sparse and should be considered after consensus with the members of the MDT and patient’s preferences.

A past medical history of melanoma is crucial as it is likely to continue to develop in an insidious manner, manifesting as a variety of symptoms, including obstructive jaundice.

## Data Availability

Data is available upon request due to privacy/ethical considerations.
